# Regulation of microRNA‐221, ‐222, ‐21 and ‐27 in articular cartilage subjected to abnormal compressive forces

**DOI:** 10.1113/JP279810

**Published:** 2020-10-31

**Authors:** Paulina S. Stadnik, Sophie J. Gilbert, Jessica Tarn, Sarah Charlton, Andrew J. Skelton, Matthew J. Barter, Victor C. Duance, David A. Young, Emma J. Blain

**Affiliations:** ^1^ Biomechanics and Bioengineering Research Centre Versus Arthritis Biomedicine Division School of Biosciences The Sir Martin Evans Building Cardiff University Cardiff Wales UK; ^2^ Skeletal Research Group Institute of Genetic Medicine Newcastle University Newcastle upon Tyne UK

**Keywords:** articular cartilage, *CPEB3*, mechanical load, miRNA, *TIMP3*

## Abstract

**Key points:**

- microRNAs (miRs) are small non‐coding molecules that regulate post‐transcriptional target gene expression.
- miRs are involved in regulating cellular activities in response to mechanical loading in all physiological systems, although it is largely unknown whether this response differs with increasing magnitudes of load.
- miR‐221, miR‐222, miR‐21‐5p and miR‐27a‐5p were significantly increased in *ex vivo* cartilage explants subjected to increasing load magnitude and in *in vivo* joint cartilage exposed to abnormal loading.
- *TIMP3* and *CPEB3* are putative miR targets in chondrocytes
- Identification of mechanically regulated miRs that have potential to impact on tissue homeostasis provides a mechanism by which load‐induced tissue behaviour is regulated, in both health and pathology, in all physiological systems.

**Abstract:**

MicroRNAs (miRs) are small non‐coding molecules that regulate post‐transcriptional target gene expression and are involved in mechano‐regulation of cellular activities in all physiological systems. It is unknown whether such epigenetic mechanisms are regulated in response to increasing magnitudes of load. The present study investigated mechano‐regulation of miRs in articular cartilage subjected to ‘physiological’ and ‘non‐physiological’ compressive loads *in vitro* as a model system and validated findings in an *in vivo* model of abnormal joint loading. Bovine full‐depth articular cartilage explants were loaded to 2.5 MPa (physiological) or 7 MPa (non‐physiological) (1 Hz, 15 min) and mechanically‐regulated miRs identified using next generation sequencing and verified using a quantitative PCR. Downstream targets were verified using miR‐specific mimics or inhibitors in conjunction with 3′‐UTR luciferase activity assays. A subset of miRs were mechanically‐regulated in *ex vivo* cartilage explants and *in vivo* joint cartilage. miR‐221, miR‐222, miR‐21‐5p and miR‐27a‐5p were increased and miR‐483 levels decreased with increasing load magnitude. Tissue inhibitor of metalloproteinase 3 (*TIMP3*) and cytoplasmic polyadenylation element binding protein 3 (*CPEB3*) were identified as putative downstream targets. Our data confirm miR‐221 and ‐222 mechano‐regulation and demonstrates novel mechano‐regulation of miR‐21‐5p and miR‐27a‐5p in *ex vivo* and *in vivo* cartilage loading models. *TIMP3* and *CPEB3* are putative miR targets in chondrocytes. Identification of specific miRs that are regulated by increasing load magnitude, as well as their potential to impact on tissue homeostasis, has direct relevance to other mechano‐sensitive physiological systems and provides a mechanism by which load‐induced tissue behaviour is regulated, in both health and pathology.

## Key points

- microRNAs (miRs) are small non‐coding molecules that regulate post‐transcriptional target gene expression.
- miRs are involved in regulating cellular activities in response to mechanical loading in all physiological systems, although it is largely unknown whether this response differs with increasing magnitudes of load.
- miR‐221, miR‐222, miR‐21‐5p and miR‐27a‐5p were significantly increased in *ex vivo* cartilage explants subjected to increasing load magnitude and in *in vivo* joint cartilage exposed to abnormal loading.
- *TIMP3* and *CPEB3* are putative miR targets in chondrocytes
- Identification of mechanically regulated miRs that have potential to impact on tissue homeostasis provides a mechanism by which load‐induced tissue behaviour is regulated, in both health and pathology, in all physiological systems.

## Introduction

Mechanical loading is essential with respect to regulating the functional capabilities of physiological systems including the musculoskeletal, cardiovascular and nervous system; this is achieved, at the cell and tissue level, by adapting to changes in mechanical load and/or metabolic stress applied. One of the major musculoskeletal tissues, articular cartilage, primarily functions to dissipate mechanical forces across the synovial joint surface and facilitates smooth, low‐friction movement. The biomechanical integrity of articular cartilage is reliant on the biochemical composition of the extracellular matrix (ECM) (Gilbert & Blain, 2018), and maintenance of cartilage tissue homeostasis, effected by the chondrocytes, is similarly dependent on mechanical load (Buckwalter *et al*. 2005). Joint articular cartilage is predominantly exposed to dynamic compressive forces, although both tensile strain and shear stresses also result from everyday movement (Lee *et al*. 2005). Application of moderate, physiological mechanical loads is essential for maintaining cartilage homeostasis by promoting anabolic activities such as increased production of ECM molecules, whereas abnormal, non‐physiological joint loading, as characterized by either overload or insufficient load, disrupts the homeostatic balance, favouring catabolism and cartilage degeneration, comprising the hallmark of osteoarthritis (OA) (Felson, 2013).

Mechano‐regulation of cellular activities within physiological systems is known to occur through epigenetic mechanisms (e.g. RNA silencing). Primary contributors to RNA silencing are the microRNAs (miR), which are small (20–2 3bp), non‐coding cytoplasmic RNAs that control the post‐transcriptional regulation of one‐third of all genes and are important in development, homeostasis and degeneration of tissues, including articular cartilage (Goldring & Marcu, 2012). Epigenetic studies have demonstrated that mechanical force has an impact on cellular responses through regulation of miR expression levels in tendon fibroblasts (Mendias *et al*. 2012), smooth muscle cells (Song *et al*. 2012), trabecular meshwork cells (Luna *et al*. 2011) and endothelial cells (Qin *et al*. 2010; Weber *et al*. 2010; Zhou *et al*. 2011). A small number of miRs were also identified as being mechanosensitive in chondrocytes (Dunn *et al*. 2009; Guan *et al*. 2011; Jin *et al*. 2014; Yang *et al*. 2016; Cheleschi *et al*. 2017). However, these studies were performed on isolated cells devoid of a substantial ECM, a feature known to be critical for cell–matrix mechano‐communications (Guilak *et al*. 2006).

Therefore, using articular cartilage as a model system, the present study aimed to identify miRs that respond to ‘physiological’ and ‘non‐physiological’ mechanical loads and to investigate the regulation of their potential downstream target genes.

## Materials and methods

Reagents were from Sigma (Poole, UK) unless otherwise specified; molecular biology reagents and plastic ware were certified RNase and DNase‐free. Culture medium consisted of Dulbecco's modified Eagle's medium/Ham's F12‐glutamax (1:1; Life Technologies, Paisley, UK) supplemented with 100 μg mL^−1^ penicillin, 100 U mL^−1^ streptomycin, 50 μg mL^−1^ ascorbate‐2‐phosphate and 1 × insulin‐transferrin‐selenium ethanolamine (1 × ITS‐X; Life Technologies).

### Preparation of articular cartilage explants and high‐density primary chondrocytes

Full depth articular cartilage explants were removed (5 mm biopsy punch; Selles Medical Limited, Hull, UK) from the metacarpophalangeal joint of <3‐week old bovine calves within 6 h of slaughter (F. Drury & Sons Abattoir, Swindon, UK); ethical approval was not required. Cartilage explants were equilibrated in culture medium for 3 days prior to mechanical load. Primary chondrocytes were isolated from full depth cartilage utilizing the same tissue source as explants, and enzymatic digestion was performed (Al‐Sabah *et al*. 2016). All cultures were maintained in 5% CO_2_ and 20% O_2_ at 37°C. Each experiment utilized tissue from between two and three animals, and repeat experiments utilized tissue from independent animals.

### *In vitro* application of mechanical load to cartilage explants

Cartilage explants were subjected to either a ‘physiological’ (2.5 MPa, 1 Hz) or a ‘non‐physiological’ load (7 MPa, 1 Hz) for 15 min using the ElectroForce 3200 (TA Instruments, New Castle, DE, USA) (Al‐Sabah *et al*. 2016) and gene expression analysed at 2, 6 and 24 h post‐load; unloaded explants served as controls. Explants were immediately snap frozen and remained in liquid nitrogen (<48 h) until RNA extraction. Loading regimes were selected based on articular cartilage literature demonstrating that ≤5 MPa is generally accepted as a ‘physiological’ load (Grodzinsky *et al*. 2000; Fehrenbacher *et al*. 2003), whereas peak loads >5 MPa are considered degradative (i.e. ‘non‐physiological’) (Fehrenbacher *et al*. 2003); the frequency was set at 1 Hz, which has been demonstrated to resemble a human fast walking speed (Bader *et al*. 2011).

### *In vivo* application of mechanical load

Twelve‐week old male C57Bl6 mice (∼25 g; Envigo, Huntington, UK) were randomly assigned to either experimental or control groups and randomly allocated to MB1 cages (960 cm^2^) in groups of five (12:12 h light/dark photocycles, with food and water available *ad libitum*). Animal husbandry and procedures were performed in compliance with the Animals (Scientific Procedures) Act 1986 [Home Office licence P287E87DF] according to Home Office and ARRIVE guidelines (Kilkenny *et al*. 2010). Mice were anaesthetized with isoflurane and custom‐built cups used to hold the right ankle and knee in flexion with a 30^o^ offset prior to the application of a 0.5 N pre‐load (ElectroForce13200; TA Instruments, Elstree, UK). A single 12 N load at a velocity of 1.4 mm s^−1^ was then applied resulting in anterior cruciate ligament (ACL) rupture as described previously (Gilbert *et al*. 2018); mechanical loading was always conducted in the morning. Buprenorphine (0.05 mg kg^−1^) was administered s.c. to mice at the start of the experiment; animals were able to move freely and were monitored for welfare and lameness until termination of the experiment. Mice were culled by cervical dislocation at either day 1 or 7 post‐load and the knee articular cartilage was dissected and processed for histology (toluidine blue staining) as described previously (Gilbert *et al*. 2018) or immediately snap frozen and remained in liquid nitrogen until RNA extraction. These early time points allowed assessment of mechanically regulated miRs in cartilage prior to overt degenerative changes and ECM loss. Nine animals were utilized for quantification of miR levels and the representative histology depicting the loading model phenotype is derived from experiments published in Gilbert *et al*. (2018).

### RNA extraction and reverse transcription for mRNA analysis

Total RNA was extracted from cartilage explants/chondrocytes using 500 μL of Trizol reagent (Invitrogen, Paisley, UK) (Al‐Sabah *et al*. 2016). RNA integrity was assessed (2100 Bioanalyzer and associated RNA 6000 Nano kit; Agilent Technologies, Wokingham, UK) and RNA integrity numbers >8.5 were observed. cDNA (total volume of 20 μL) was synthesized from 300 ng of total RNA using Superscript III reverse transcriptase in conjunction with 0.5 μg of random primers (Promega, Southampton, UK) in accordance with the manufacturer's instructions (Invitrogen).

### RNA extraction and reverse transcription for miR analysis

Total RNA was extracted from cartilage explants/chondrocytes as described above, except 1 mL of Trizol reagent was used. After ethanol precipitation, total RNA was purified using a mirVana miR Isolation Kit (Ambion, Paisley, UK) in accordance with the manufacturer's instructions. RNA integrity numbers of >8.0 were observed. cDNA of mature miRs was generated separately from total RNA (5 ng) using the TaqMan MicroRNA Reverse Transcription Kit (Applied Biosystems, Paisley, UK) involving 50 U of MultiScribe Reverse Transcriptase and stem‐looped reverse transcription primers, specific to individual miRs, from TaqMan MicroRNA Assays (Applied Biosystems, Paisley, UK) in accordance with the manufacturer's instructions.

### miR next generation sequencing and bioinformatic analysis

Mechanically‐regulated articular cartilage miRs were identified using next generation sequencing (NGS) using >3.5 μg of RNA per sample. Procedures were conducted in accordance with the manufacturers’ instructions. Library preparation was conducted on 450 ng of total RNA using the NEB Next Small RNA Library Prep Set for Illumina (Multiplex Compatible: BioLabs, Hitchin, UK) and amplified cDNA was purified using a QIAquick PCR Purification Kit (Qiagen, Crawley, UK). miR libraries were selected by running purified cDNA samples on 8% (v/v) polyacrylamide gels and excising bands located at ∼140 bp (Crowe *et al*. 2016). A Multiplex Compatible kit (NEB Next Small RNA Library Prep Set for Illumina) was used to elute and purify the miRs, and the concentration of miR libraries assessed prior to analysis on a HiSeq Sequencing System (The Genome Analysis Centre, Norwich, UK). miR deep sequencing data (raw FASTQ files) were run through FastQC and Cutadapt (Martin 2011), and trimmed FASTQ files were aligned against known *bos taurus* miR sequences from miRBase (http://www.mirbase.org). Quantification was determined by counting aligned reads against a reference, using a combination of RSamTools and ShortRead (Li *et al*. 2009) bioconductor packages. Differential expression was assessed using DESeq2 (Love *et al*. 2014). Global experimental variance was analysed using principal component analysis to assess for outlier samples and statistical significance from differential expression tests was determined by retaining miRs that had an adjusted *P* < 0.05.

### Manipulation of miR expression levels in high‐density chondrocyte cultures

Primary bovine chondrocytes were seeded onto six‐well culture plates (VWR, Lutterworth, UK) at a density of 4 × 10^6^ cells per well in antibiotic‐free culture media and incubated at 37°C for 24 h prior to transfection. Chondrocytes were transfected for 48 h with 50 nm mirVana miR inhibitors (Applied Biosystems) or 50 nm miScript miR mimics (Qiagen) using DharmaFECT1 lipid reagent (Dharmacon, Cambridge, UK) in accordance with the manufacturer's instructions; mirVana miR Inhibitor Negative Control #1 (Applied Biosystems) and AllStars negative control small interfering RNA (siRNA) (Qiagen) were utilized as transfection controls (50 nm).

### Quantification of miRNA and mRNA transcripts

Quantification of mRNA or miR in experimental samples was performed using a MxPro3000 QPCR system (Agilent Technologies, Stockport, UK) and measured using either reference gene primers (MWG‐Biotech AG, Ebersberg, Germany) or bovine‐specific TaqMan probes (Applied Biosystems, Paisley, UK) in conjunction with either Brilliant III Ultra‐Fast SYBR Green QPCR Master Mix (Agilent Genomics, Berkshire, UK) or TaqMan Fast Advanced Master Mix (Applied Biosystems, Paisley, UK). Reference gene primers (200 nM final concentration (Al‐Sabah *et al*. 2016)) including SDHA, YWHAZ, HPRT, 18 s and β‐actin were validated as per MIQE guidelines (Bustin *et al*. 2009). Cycling conditions were: 95°C‐3 min (1 cycle), 95°C‐15 s followed by 60°C‐30 s (40 cycles) with an additional dissociation cycle of 95°C‐1min, 60°C‐30 s followed by 95°C‐30 s (1 cycle) to confirm primer specificity with SYBR Green detection. Relative quantification was calculated using the 2^−ΔΔ^CT method (Livak & Schmittgen, 2001), with unloaded controls as a reference group to quantify relative changes in transcript expression. Fold change was normalized to the geometric mean of 2–3 reference genes whose expression was identified as stable under the experimental condition using RefFinder software (https://www.heartcure.com.au/for-researchers/).

### Luciferase activity assays

The 3′‐UTR of mRNAs, containing the predicted binding site of target miRs, were cloned into pmirGLO dual‐luciferase miRNA target expression vector (Promega, Southampton, UK) by In‐Fusion (Takara Bio Europe, Saint‐Germain‐en‐Laye, France) and construct sequences verified (for primer sequences, see Table 1). SW1353 chondrosarcoma cells (∼20 000 cells cm^–2^) were co‐transfected with 50 nm miRNA mimics with the reporter plasmids (500 ng mL^−1^) (Barter *et al*. 2015); transfection of 50 nm AllStars negative control siRNA with the reporter plasmids was used as control. Following a 24 h transfection, cells were lysed and luciferase levels were determined using a Promega GloMax luminometer and the Dual‐Luciferase reporter assay system (Promega).

**Table 1:** Sequences of primers used to clone the 3′‐UTR of mRNAs containing the predicted binding site of target miRs

Target	5′‐ to 3′ Oligo sequence	Annealing temperature (°C)
*TIMP3* UTR	F 5′‐GCTCGCTAGCCTCGACTGAGCTTCCCTTGGACACT‐3′ R 5′‐CGACTCTAGACTCGAGCTAAAGGGAAAGGCGGAT‐3′	60
*CPEB3* UTR	F 5′‐GCTCGCTAGCCTCGAAAGGAGGGAAAAGAGAGGGC‐3′ R 5′‐CGACTCTAGACTCGAAACAGAGCACCGCAAAGTAC‐3′	60

### Statistical analysis

Quantitative PCR (qPCR) data are presented as the mean ± 95% confidence intervals (CIs) after normalization to identified reference genes for explants (*SDHA* and *YWHAZ*), transfected cells (*HPRT* and *YWHAZ*) or *in vivo* model (*U6*, *18s* and *β‐actin*) and further normalized to untreated controls. Experiments were performed on explants (*n* = 6), transfected cells (*n* = 3) and *in vivo* studies (*n* = 9), with three independent repeats for explant and cell studies. Data were assessed for normality and differences in variances and transformed where required. One‐way ANOVA and Fisher's *post hoc* test were performed to determine significance of mechanical load or manipulation of miR expression levels on gene expression, respectively; the results were considered statistically significant at *P* < 0.05 (Minitab, version 17; Minitab, LLC, State College, PA, USA).

## Results

### Identification and validation of mechanically‐regulated miRs in cartilage explants

**Figure 1:**
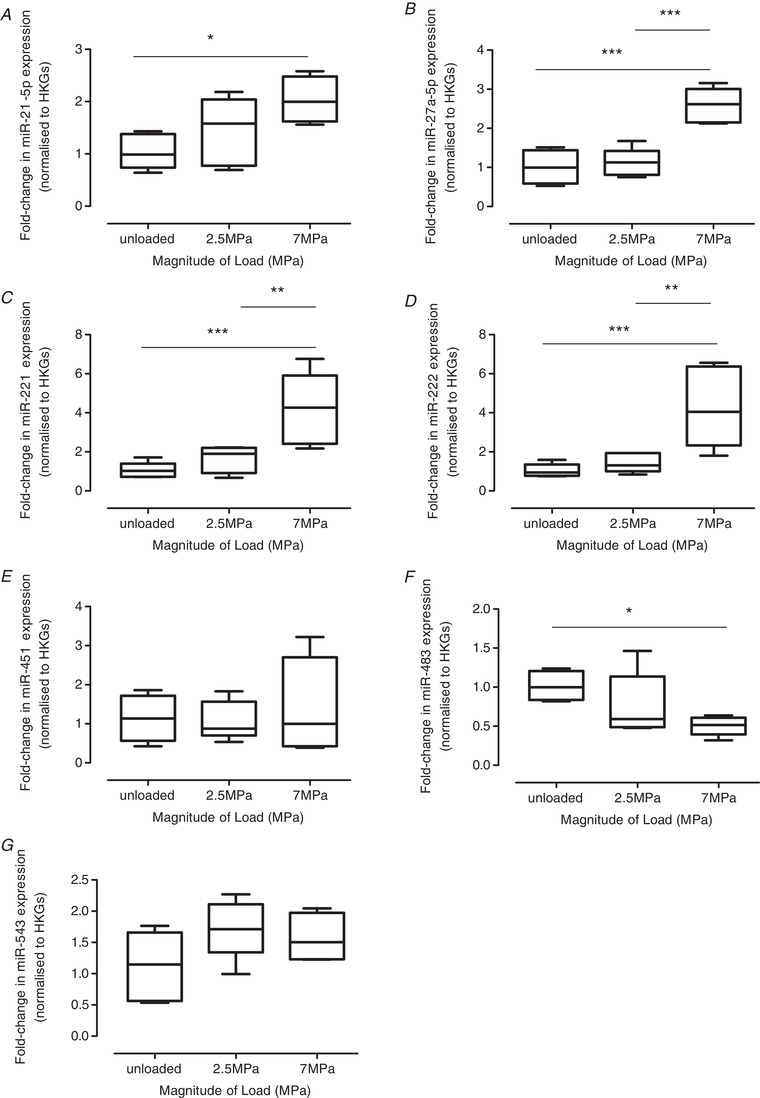
Validation of mechanically‐regulated miRs in cartilage explants using qPCR qPCR validation of mechanically‐regulated miRs, identified by NGS, in cartilage explants subjected to loads of 2.5 or 7 MPa (1 Hz, 15 min) and analysed 24 h post‐cessation of load for (*A*) miR‐21‐5p, (*B*) miR‐27a‐5p, (*C*) miR‐221, (*D*) miR‐222, (*E*) miR‐451, (*F*) miR‐483 and (*G*) miR‐453; unloaded explants served as controls. miR levels were normalized to the geometric mean of two reference genes (*SDHA*, *YWHAZ*) and further normalized relative to the unloaded control cDNAs. Data are presented as box plots depicting the mean ± 95% CI (*n* = 6 explants) and are representative of three independent experiments. Statistical analysis was performed using one‐way ANOVA with Tukey's *post hoc* test.

NGS was performed on cartilage explants subjected to a 2.5 or 7 MPa load (1 Hz, 15 min) to identify mechano‐sensitive miRs (Table 2); unloaded explants served as controls. NGS analysis was conducted at 2, 6 or 24 h post‐load to investigate temporal differences in miR expression. A small number of annotated miRs were significantly regulated by load at 2 h (8 miRs) (Table 2) and 6 h (5 miRs) (Table 2), with 17 miRs detected at 24 h post‐load (Table 2). A greater number of miRs were only regulated by the non‐physiological 7 MPa load (Table 2). Given the number of significant changes at 24 h post‐load (Table 2), the top five most significantly up‐regulated (i.e. miR‐222, ‐27a‐5p, ‐221, ‐543 and ‐21‐5p) and the two most significantly down‐regulated (i.e. miR‐451 and ‐483) were validated for this time point using TaqMan qPCR on individual RNA samples (Fig. 1). Relative to unloaded, a 7 MPa load increased expression of miR‐21‐5p (two‐fold; *P* = 0.034) (Fig. 1 *A*), miR‐27‐5p (2.56‐fold; *P* = 0.001) (Fig. 1 *B*), miR‐221 (3.85‐fold; *P* < 0.001) (Fig. 1 *C*) and miR‐222 (3.78‐fold; *P* < 0.001) (Fig. 1 *D*), and decreased miR‐483 expression (two‐fold; *P* = 0.047) (Fig. 1 *F*). A loading magnitude‐dependent regulation of miRs was observed between explants subjected to a 7 MPa *vs*. 2.5 MPa load: miR‐27‐5p (2.4‐fold, *P* < 0.001) (Fig. 1 *B*), miR‐221 (2.55‐fold, *P* = 0.011) (Fig. 1 *C*) and miR‐222 (2.83‐fold, *P* = 0.002) (Fig. 1 *D*). Although miR‐seq indicated that a 7 MPa load down‐regulated miR‐451 levels (2.1‐fold, *P* = 0.002) (Table 2) and up‐regulated miR‐543 levels (2.62‐fold, *P* < 0.001; *P* = 0.005) (Table 2) relative to unloaded cartilage, this was not verified using qPCR (Fig. 1 *E* and *G*).

**Table 2:** Mean fold‐change and statistical significance of mechanically‐regulated miRs in articular chondrocytes subjected to loads of 2.5 or 7 MPa (1 Hz, 15 min), to represent a physiological or non‐physiological load respectively, and analysed 2, 6 and 24 h post‐cessation of load (unloaded explants served as controls)

	UL *vs*. 2.5MPa		UL *vs*. 7MPa		2.5 *vs*. 7MPa	
	*FC*	*P*adj	*FC*	*P*adj	*FC*	*P*adj
Analysed 2 h post‐cessation of load						
miR‐27a‐5p	4.420	2.79 × 10^–22^	7.092	3.08 × 10^–40^		
miR‐2898	0.588	0.008	0.408	3.16 × 10^–10^		
miR‐2478			0.566	0.001		
miR‐98			1.698	0.001		
miR‐23b‐3p			1.539	0.004		
miR‐1260b			0.647	0.025		
miR‐23a			1.464	0.034		
miR‐148b			1.644	0.039		
Analysed 6 h post‐cessation of load						
miR‐486	0.521	0.002				
miR‐677			2.149	5.50 × 10^–5^	1.933	0.001
miR‐222			1.568	0.008		
miR‐2889					2.393	1.54 × 10^–6^
miR‐1249					0.562	0.013
Analysed 24 h post‐cessation of load						
miR‐222	1.814	0.003	7.409	8.81 × 10^–51^	4.085	4.00 × 10^–25^
miR‐27a‐5p			3.019	1.14 × 10^–16^	2.027	5.84 × 10^–7^
miR‐221			3.393	1.63 × 10^–13^	2.593	3.58 × 10^–8^
miR‐543			2.619	1.30 × 10^–9^	1.725	0.005
miR‐21‐5p			2.267	6.67 × 10^–6^	1.719	0.013
miR‐495			1.775	1.45 × 10^–4^		
miR‐451			0.481	0.002		
miR‐425‐5p			0.626	0.010	0.672	0.037
miR‐20a			1.603	0.012		
miR‐7			1.699	0.017		
miR‐760‐3p			1.715	0.017		
miR‐2318			1.8518	0.022		
miR‐2344			1.829	0.030		
miR‐431			1.817	0.030		
miR‐155			1.472	0.042		
miR‐100			1.429	0.048		
miR‐483					0.599	0.025

Data are representative of three independent experiments (*n* = 6 explants per individual experiment).

### *In vivo* validation of miR‐21‐5p, miR‐27‐5p, miR‐221 and miR‐222 mechano‐regulation

**Figure 2:**
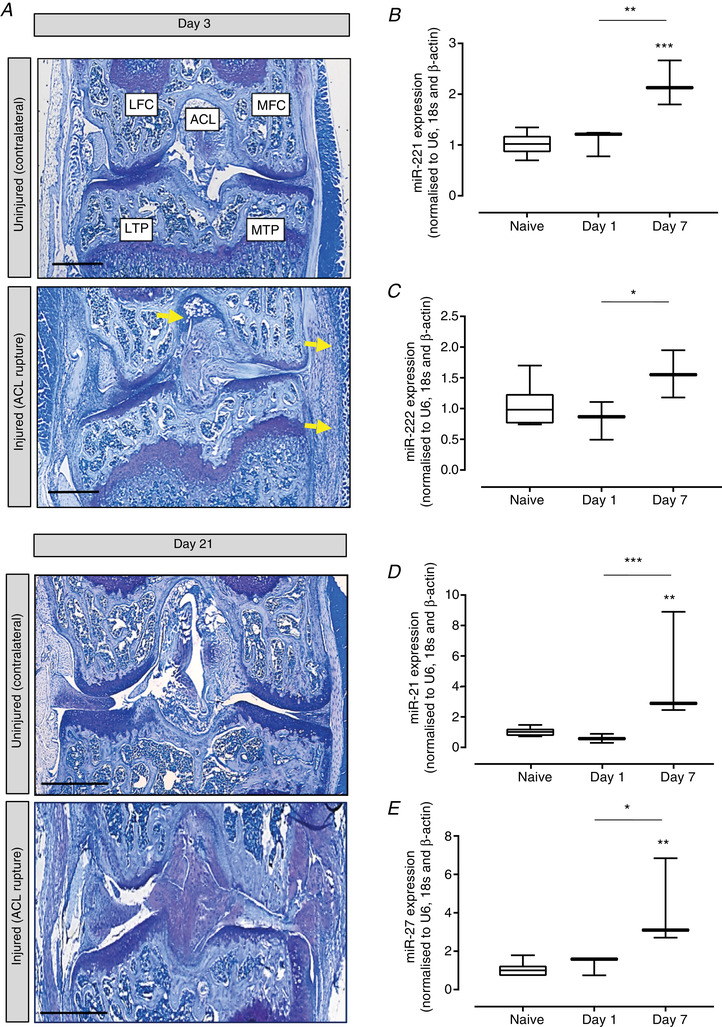
Validation of mechanically regulated miRNAs in a murine *in vivo* model of load‐induced joint degeneration *A*, toluidine blue staining of a representative mouse knee joint at days 3 and 21 after ACL rupture to induce joint instability/joint degeneration. MTP, medial tibial plateau; MFC, medial femoral condyle; LTP, tibial plateau; LFC, lateral femoral condyle; ACL, anterior cruciate ligament. Yellow indicates inflammatory cell infiltrate. Validation of differential expression of (*B*) miR‐221, (*C*) miR‐222, (*D*) miR‐21‐5p and (*E*) miR‐27‐5p in articular cartilage after normalization to the geometric mean of the reference genes *U6*, *β‐actin* and *18s* and further normalization to the uninjured knee cartilage. Data are presented as box plots depicting the mean ± 95% CI (*n* = 3 animals per experimental time point). Statistical analysis was performed using one‐way ANOVA with Tukey's *post hoc* test. [Color figure can be viewed at wileyonlinelibrary.com]

The physiological relevance of identified mechanically‐regulated miRs was determined in a murine *in vivo* model of post‐traumatic OA, in which ACL rupture induces mechanical instability, by applying an abnormal load to the knee joint (Gilbert *et al*. 2018). Synovial infiltration occurs rapidly followed by extensive joint degeneration as characterized by articular cartilage loss and bone remodelling by day 21 (Fig. 2 *A*) (Gilbert *et al*. 2018). However, at the earlier stages analysed in the present study, the articular cartilage is intact. Of the original miRs identified (Table 1), four that were successfully validated by qPCR (miR‐221, ‐222, ‐21‐5p and ‐27‐5p) (Fig. 1) were subsequently analysed *in vivo*. No significant effects were detected after 1 day of destabilization; however, after 7 days of mechanical instability, miR‐221 (2.20‐fold, *P* < 0.001) (Fig. 2 *B*), miR‐222 (1.56‐fold, *P* = 0.070) (Fig. 2 *C*), miR21‐5p (4.75‐fold, *P* = 0.002) (Fig. 2 *D*) and miR‐27‐5p (4.21‐fold, *P* = 0.003) (Fig. 2 *E*) were up‐regulated compared to naïve mice (control). Furthermore, miR‐221 (2.05‐fold, *P* = 0.003) (Fig. 2 *B*), miR‐222 (1.90‐fold, *P* = 0.030) (Fig. 2 *C*), miR21‐5p (7.99‐fold, *P* = 0.001) (Fig. 2 *D*) and miR‐27‐5p (3.19‐fold, *P* = 0.013) (Fig. 2 *E*) were all significantly up‐regulated compared to mice after 1 day of joint instability.

### miR target gene validation

**Figure 3:**
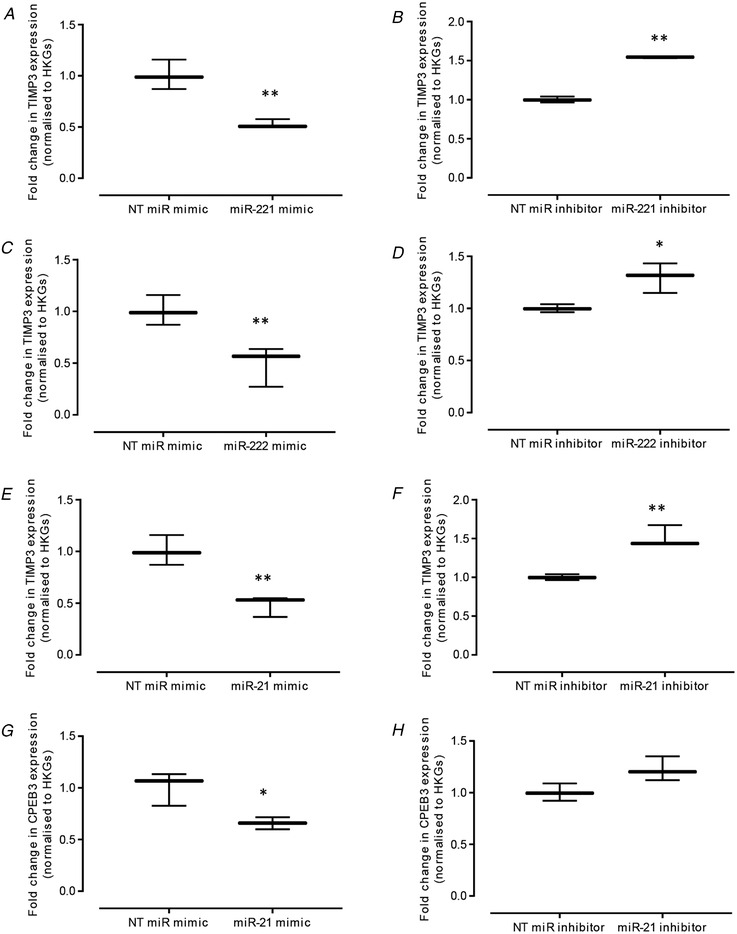
Validation of *TIMP3* and *CPEB3*, putative target genes of miR‐221, miR‐222 or miR‐21, using TaqMan qPCR Primary bovine chondrocytes were treated with either 50 nm miR mimic, 50 nm inhibitor or negative control siRNAs for each respective miR for 48 h, prior to analysis of the effect of overexpression and knockdown of miR‐221 on *TIMP3* transcription (*A* and *B*), miR‐222 on *TIMP3* transcription (*C* and *D*), and miR‐21 on *TIMP3* (*E* and *F*) and *CPEB3* (*G* and *H*) transcript levels after normalization to the geometric mean of the reference genes *HPRT* and *YWHAZ* and further normalization to respective negative control siRNAs. Data are presented as the mean ± 95% CI (*n* = 3 wells) and are representative of three independent experiments. Statistical analysis was performed using one‐way ANOVA with Tukey's *post hoc* test.

Potential miR target genes identified by NGS were determined using Targetscan (http://www.targetscan.org) in conjunction with an assessment of their relevance to mechanical load or cartilage homeostasis as determined using the literature; putative target genes were examined by manipulation of expression levels using specific miR mimics or inhibitors (Fig. 3). Three putative miR‐21‐5p targets were selected: cytoplasmic polyadenylation element binding protein 3 (*CPEB3*), matrix metalloproteinase 13 (*MMP13*) and tissue inhibitor of metalloproteinase 3 (*TIMP3*). miR‐221 and miR‐222 seed sites are identical; hence, the selected putative target genes included: *CPEB3*, leukaemia inhibitory factor receptor (*LIFR*) and *TIMP3*. miR‐27a target gene validation was not performed because a consistent reduction in miR‐27a expression was not achieved using specific antagomirs. qPCR analysis confirmed that mimic‐induced elevations in miR‐221 levels resulted in a significant reduction in *TIMP3* (*P* = 0.006) (Fig. 3 *A*). Conversely, inhibition of miR‐221 expression correlated with a significant increase in *TIMP3* transcription (*P* = 0.003) (Fig. 3 *B*). Similarly, a mimic‐induced increase in miR‐222 levels led to a significant reduction in *TIMP3* (*P* = 0.006) (Fig. 3 *C*). Conversely, inhibition of miR‐222 expression correlated with a significant increase in *TIMP3* (*P* = 0.025) (Fig. 3 *D*). No other putative target genes were robustly regulated by miR‐221 or 222. Mimic‐induced miR‐21 levels resulted in a significant reduction in *TIMP3* (*P* = 0.006) (Fig. 3 *E*) and *CPEB3* transcription (*P* = 0.015) (Fig. 3 *G*). Conversely, inhibition of miR‐21 expression correlated with a significant increase in *TIMP3* (*P* = 0.010) (Fig. 3 *F*) and no significant effect on *CPEB3* (*P* = 0.068) (Fig. 3 *H*). *MMP13* expression was not consistently regulated by miR‐21 (data not shown).

**Figure 4:**
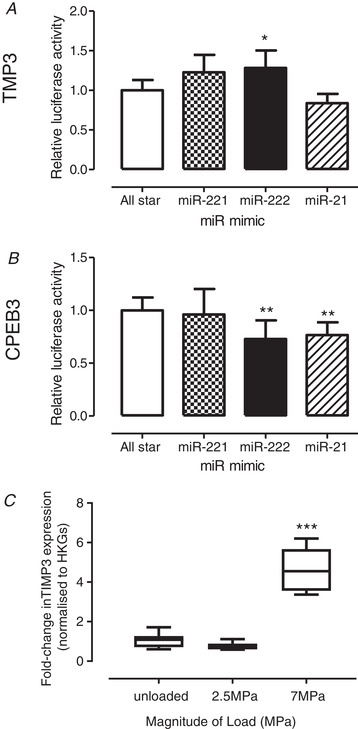
Verification of 3′‐UTR activation of target mRNAs containing the predicted miR seed sites using a luciferase promoter assay SW1353 chondrosarcoma cells were co‐transfected with reporter plasmids containing either (*A*) *TIMP3* or (*B*) *CPEB3* 3′‐UTRs and 50 nm miR‐221, miR‐222 or miR‐21‐5p mimics, or the negative control siRNA, for 24 h and luciferase levels were determined. Data are presented as the mean ± 95% CI (*n* = 3 wells) and are representative of three independent experiments. *C*, *TIMP3 m*RNA levels, as assessed using qPCR, in cartilage explants subjected to loads of 2.5 or 7 MPa (1 Hz, 15 min) and analysed 24 h post‐cessation of load; unloaded explants served as controls. mRNA levels were normalized to the geometric mean of two reference genes (*SDHA*, *YWHAZ*) and further normalized relative to the unloaded control cDNAs. Data are presented as box plots depicting the mean ± 95% CI (*n* = 6 explants) and are representative of three independent experiments. Statistical analysis was performed using one‐way ANOVA with Tukey's *post hoc* test.

Activation of the 3′‐UTR of target mRNAs containing the predicted seed sites was investigated (Fig. 4). Addition of miR‐21 mimic significantly suppressed luciferase activity regulated by *TIMP3* (*P* = 0.053) (Fig. 4 *A*) and Cpeb3 3′‐UTRs (*P* = 0.010) (Fig. 4 *B*). Furthermore, miR‐222 mimic also significantly inhibited *CPEB3* 3′‐UTR regulated luciferase activity (*P* = 0.010) (Fig. 4 *B*). Surprisingly, and in contrast to qPCR validation, miR‐222 overexpression significantly increased luciferase activity regulated by *TIMP3* 3′‐UTR (*P* = 0.030) (Fig. 4 *A*). Although this contradicts the miR‐222 mimic data demonstrating a significant *TIMP3* reduction (Fig. 3 *C*), it does substantiate the load‐induced *TIMP3* observed in the *in vitro* loading model where *TIMP3* transcription was elevated in response to 7 MPa load (4.8‐fold, *P* < 0.001) (Fig. 4 *C*).

## Discussion

Physiological forces are critical for maintaining tissue homeostasis, and the involvement of epigenetic mechanisms such as mechano‐regulation of miR expression occurs in many tissues, including articular cartilage (Dunn *et al*. 2009; Guan *et al*. 2011; Jin *et al*. 2014; Yang *et al*. 2015; Cheleschi *et al*. 2017). However, our understanding of miR involvement in response to different magnitudes of mechanical forces and, specifically, its impact on controlling mechanically induced tissue homeostasis is still in its infancy. The present study investigated the mechano‐regulation of miRs in articular cartilage tissue explants subjected to ‘physiological’ and ‘non‐physiological’ loads *in vitro* and validated regulated miRs in a murine *in vivo* model of abnormal joint loading. In addition, the study identified downstream miR targets to provide insight on mechanisms of mechanically mediated cartilage homeostasis. Importantly, the seed regions of the miRs of interest analysed in the present study are evolutionarily conserved across bovine, mouse and human species, indicating their potential physiological relevance.

Analysis of the miR‐seq data illustrated that (i) a miR‐mediated response to a 15 min loading episode was most noticeable at 24 h post‐load and (ii) differentially regulated miRs were largely responsive to non‐physiological compressive loads; the small number of miRs that were significantly regulated in response to physiological load probably reflects the loading regimen period. The miRs that were identified and validated to be most robustly altered by non‐physiological load compared to unloaded controls and to physiological load were miR‐221 and miR‐222. This confirms the mechano‐sensitive nature of miR‐221 and miR‐222, previously shown in cardiomyocytes after cardiac overload (El‐Armouche *et al*. 2010), as well as in tendon fibroblasts (Mendias *et al*. 2012), engineered cartilage constructs in response to a catabolic loading regimen (Hecht *et al*. 2019) and anterior weight‐bearing cartilage relative to the posterior non‐weight bearing tissue in bovine stifle joints (Dunn *et al*. 2009).

Chondrogenic markers *COL2A1* and *SOX9 *have been identified as putative gene targets for miR‐221 and miR‐222 (conserved seed site) that may influence cartilage homeostasis (Lolli *et al*. 2014); furthermore, miR‐221 silencing strongly enhanced *in vivo* cartilage repair (Lolli *et al*. 2016). miR‐221 inhibition also enhanced expression of chondrocyte‐like phenotypic markers in intervertebral disc cells (Penolazzi *et al*. 2018). Therefore, miR‐221 and miR‐222 induction, observed in the cartilage explants in response to non‐physiological load (Al Sabah A., Duance V. C., Blain E. J., unpublished observations), suggests an attempt to remodel the cartilage tissue to confer a more appropriate biomechanical response.

Analysis of downstream target genes identified robust regulation of *TIMP3* only. However, although *TIMP3* was clearly regulated via overexpression/inhibition studies in primary chondrocytes, this did not reflect observations in the SW1353 chondrosarcoma cell line for 3′‐UTR activity using the luciferase assay or recapitulate events in tissue demonstrating that other, as yet unidentified, targets are regulated by miR‐221 and miR‐222 to elicit effects. These conflicting findings may be explained by the different experimental systems used in the present study, thus potentially masking the effects of other regulatory contributors with respect to the influence of miR‐221 and miR‐222 on Timp3 expression. Another possibility that might explain the simultaneous elevation of both the tested miRs and *TIMP3* is a regulatory loop , such that elevated *TIMP3* expression induces higher expression of these miRs to reduce Timp3 transcript levels in cells over time. Analysis at time points beyond 24 h post‐load would provide insight as to whether potential regulatory loops exist.

Two other miRs robustly regulated by a magnitude‐dependent load in our *in vitro* and *in vivo* loading models were miR‐21‐5p and miR‐27a‐5p. To the best of our knowledge, this is the first report of the mechano‐regulation of these miRs in articular cartilage. However, miR‐21 mechano‐regulation occurs in other cell types; tensile strain induced miR‐21 expression in human aortic smooth muscle cells (Song *et al*. 2012), and both laminar (Weber *et al*. 2010) and oscillatory shear stress (Zhou *et al*. 2011) elevated miR‐21 levels in human umbilical vein endothelial cells. By contrast, pulsatile shear stress inhibited miR‐21 expression in these endothelial cells (Zhou *et al*. 2011), revealing the mechano‐sensitive nature of these molecules. In the present study, both *TIMP3* and *CPEB3* were identified as downstream targets of miR‐21‐5p; however, as noted previously, *TIMP3* is not negatively correlated with miR‐21‐5p levels in our model systems. Furthermore, *CPEB3* levels were not significantly regulated in the present study, indicating that, although these genes are direct targets of miR‐21‐5p in primary chondrocytes, they are not directly regulated in our models. As a result of the complexities of such signalling mechanisms in the tissue, further experiments are clearly required to determine the interplay of these miRs and their mechanistic activities in cartilage homeostasis, which both remain beyond the scope of the present study.

miR‐27a‐5p was robustly regulated by mechanical load both *in vitro* and *in vivo*. Mechano‐regulation of miR‐27a in articular cartilage is a novel finding and corroborates studies demonstrating up‐regulation of both miR‐27a and miR‐27b in endothelial cells subjected to laminar flow (Urbich *et al*. 2012) and endothelial cells exposed to cyclic tensile strain (Wang *et al*. 2017). Downstream targets of miR‐27‐5p, which are known to be regulated in *in situ* cartilage explants in response to non‐physiological load Al‐Sabah *et al*. (unpublished data), include WNT signalling molecules such as DKK2 (Tao *et al*. 2015; Wu *et al*. 2019) and sFRP1 (Wu *et al*. 2019). Future studies will explore the relationship between mechano‐sensitive miR‐27‐5p and downstream regulation of WNT signalling components in cartilage homeostasis.

A reduction in miR‐483 levels was observed in response to non‐physiological load and is the first report of its mechano‐sensitivity in articular cartilage. Its potential role in cartilage homeostasis is not well defined, with conflicting evidence suggesting anabolic (Yang *et al*. 2015) and catabolic outcomes (Xu *et al*. 2017; Wang *et al*. 2019); hence, its observed reduction in response to abnormal load may reflect an attempt at tissue remodelling.

A limitation of the present study is use of immature articular cartilage removed from underlying subchondral bone, which could influence mechano‐biological outcomes. However, to mitigate this limitation, we validated identified miRs in an *in vivo* model of abnormal joint loading to confirm their mechano‐regulation; interestingly, many of the miRs regulated by load in our *in vitro* and *in vivo* models have also been reported to be differentially expressed in OA (Tardif *et al*. 2009; Zhang *et al*. 2014; Song *et al*. 2015; Wang *et al*. 2019), lending weight to their relevance in cartilage homeostasis.

In conclusion, the loading magnitude‐dependent regulation of specific miRs identified in the present study, as well as their potential to impact on tissue homeostasis, has direct relevance to other physiological systems that are mechano‐sensitive. Furthermore, it provides a pivotal mechanism by which load‐induced tissue behaviours are regulated, in both health and pathology, and is critical to understand with respect to successful tissue engineering strategies in physiological systems.

## Additional information

### Competing interests

The authors declare that they have no competing interests.

### Author contributions

PS, VCD, DAY and EJB were responsible for study conception and design. PS, SJG, SC, JT, AJS and EJB were responsible for data acquisition and statistical analysis. PS, SJG, SC, MJB, VCD, DAY and EJB were responsible for data analysis and interpretation. EJB was responsible for manuscript preparation. PS, SJG, SC, JT, AJS, MJB, VCD, DAY and EJB were responsible for critical revision of the draft. All authors approved the final article submitted for publication and take full responsibility for integrity of the study.

### Funding

The sponsors had no role in the study design, collection, analysis and interpretation of data; in the writing of the article; nor in the decision to submit the article for publication.

## Supporting information


Statistical Summary Document

## Data Availability

The data that support the findings of this study are being made openly available in GEO (GSE158571).
