# Supplementary material for: Regulation of microRNA‐221, ‐222, ‐21 and ‐27 in articular cartilage subjected to abnormal compressive forces
Source: J Physiol. 2020 Oct 31;599(1):143–55. doi: 10.1113/JP279810 (PMC8132181; doi:10.1113/JP279810)
Supplement: Supplementary file 1 — Statistical Summary Document [file TJP-599-143-s001.docx]

| 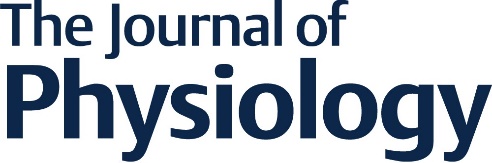   \|  \| \| --- \| |  |  |  |  |  | |  | |  |  | |  | |  | |  | |
| --- | --- | --- | --- | --- | --- | --- | --- | --- | --- | --- | --- | --- | --- | --- | --- | --- | --- | --- |
|  |  |  |  |  |  | |  | |  |  | |  | |  | |  | |
|  |  |  |  |  |  | |  | |  |  | |  | |  | |  | |
|  |  |  |  |  |  | |  | |  |  | |  | |  | |  | |
| **Statistical Summary Document** | | |  |  |  | |  | |  |  | |  | |  | |  | |
|  |  |  |  |  |  | |  | |  |  | |  | |  | |  | |
| **Manuscript Title:** | **Regulation of microRNA-221, -222, -21 and -27 in articular cartilage subjected to abnormal compressive forces.** | | | | | | | | |  |  | |  | |  | |  |
| **Authors:** | **Stadnik PS, Gilbert SJ, Tarn J, Charlton S, Skelton AJ, Barter MJ, Duance VC, Young DA, Blain EJ** | | | | |  | |  | |  |  | |  | |  | |  |
| **Animal model used, if applicable:** | **Non-surgical rupture of anterior cruciate ligament** | | |  |  | |  | |  |  | |  | |  | |  | |
| **Underlying hypothesis:** | **This study tests the hypothesis that mechano-regulation of miRNAs are differentially expressed in articular cartilage subjected to either a physiological or non-physiological compressive force** | | | | | | | | | | | | | | | |  |
| **Definitions of ‘n’:** | [Define ‘n’. If definitions differ, please indicate which definition applies to which experimental question number.] | | | | | | |  | |  |  | |  | |  | |  |
|  | **Question 1** | **n = number of individual explants from multiple animals** | | |  | |  | |  |  | |  | |  | |  | |
|  | **Question 2** | **n = number of animals (undergone non-surgical rupture of anterior cruciate ligament)** | | | | | |  | |  |  | |  | |  | |  |
|  | **Question 3 & 4** | **n = number of wells containing transfected cells** | |  |  | |  | |  |  | |  | |  | |  | |
|  |  |  |  |  |  | |  | |  |  | |  | |  | |  | |
|  |  |  |  |  |  | |  | |  |  | |  | |  | |  | |

| **Statistical summary table:** |  |  |  |  |  |  |  |  |  |  |  |  |
| --- | --- | --- | --- | --- | --- | --- | --- | --- | --- | --- | --- | --- |
|  |  |  |  |  |  |  |  |  |  |  |  |  |
|  |  |  |  |  |  |  |  |  |  |  |  |  |
| **Experimental question number** | **Finding/ conclusion** | **Experimental location/variable** | **Mean value** | **Standard Deviation** | **n** | **P** | **Units** | **Data comparisons** | **Statistical test** | **Any other variable** | **Figure/table in which data are presented** | **Comments** |
| **Multiple comparisons for the same question are shown in different rows** |  | **e.g. cortex vs cerebellum or genotype** | **(or other summary statistic)** | **(SD)** | **(value)** | **If P is considered significant against a stated confidence limit, authors may indicate this in bold** |  | **e.g. WT vs KO** |  | **e.g. subjects’ age or sex** |  | **e.g. observation** |
|  |  | Differential miR expression | > 1.5-fold | - |  | <0.05 |  | Unloaded vs 2.5MPa, unloaded vs 7MPa, 2.5MPa vs 7MPa |  |  | Table 2 |  |
| 1. Validation of identified mechanically regulated miRNAs *in vitro* | A number of miRNAs were verified as being differentially expressed 24hours post-cessation of physiological or non-physiological load in *in vitro* loading model | miR-21-5p | 2.00-fold | 0.446 | n = 6, N = 3 independent repeats | 0.034 | arbitrary expression (normalised to housekeeping genes) | unloaded vs 7MPa | One-way analysis of variance (ANOVA) and Fisher’s *post-hoc* test |  | 1A |  |
|  |  | miR-27a-5p | 2.56-fold | 0.441 |  | 0.001 |  | unloaded vs 7MPa |  |  | 1B |  |
|  |  |  | 2.40-fold | 0.358 |  | <0.001 |  | 2.5MPa vs 7MPa |  |  |  |  |
|  |  | miR-221 | 3.85-fold | 1.856 |  | <0.001 |  | unloaded vs 7MPa |  |  | 1C |  |
|  |  |  | 2.55-fold | 0.657 |  | 0.011 |  | 2.5MPa vs 7MPa |  |  |  |  |
|  |  | miR-222 | 3.78-fold | 2.002 |  | <0.001 |  | unloaded vs 7MPa |  |  | 1D |  |
|  |  |  | 2.83-fold | 0.452 |  | 0.002 |  | 2.5MPa vs 7MPa |  |  |  |  |
|  |  | miR-483 | 2.00-fold | 0.121 |  | 0.047 |  | Unloaded vs 7MPa |  |  | 1F |  |
| 2. Validation of identified mechanically regulated miRNAs *in vivo* | The most differentially regulated miRNAs were verified in an *in vivo* model of abnormal joint loading | ACL rupture joint vs contralateral control joint at day 3 and day 21 post-rupture | - | - | n = 9 animals | - | - | ACL rupture vs non-rupture joints | - | - | 2A | Observation. Representative histological images, not quantified |
|  |  | miR-221 | 2.20-fold | 0.438 |  | <0.001 | arbitrary expression (normalised to housekeeping genes) | Naïve vs Day 7 | One-way analysis of variance (ANOVA) and Fisher’s *post-hoc* test |  | 2B |  |
|  |  |  | 2.05-fold | 0.260 |  | 0.003 |  | Day 1 vs Day 7 |  |  |  |  |
|  |  | miR-222 | 1.897-fold | 0.385 |  | 0.030 |  | Day 1 vs Day 7 |  |  | 2C |  |
|  |  | miR-21-5p | 4.75-fold | 3.600 |  | 0.002 |  | Naïve vs Day 7 |  |  | 2D |  |
|  |  |  | 7.99-fold | 0.2951 |  | 0.001 |  | Day 1 vs Day 7 |  |  |  |  |
|  |  | miR-27-5p | 4.21-fold | 2.283 |  | 0.003 |  | Naïve vs Day 7 |  |  | 2E |  |
|  |  |  | 3.19-fold | 0.498 |  | 0.013 |  | Day 1 vs Day 7 |  |  |  |  |
| 3. Verification of TIMP3 or CPEB3 as a target gene | TIMP3 was verified as a target gene of the identified miRs | TIMP3 mRNA | 1.889-fold | 0.042 | n = 3, N = 3 independent repeats | 0.006 | arbitrary expression (normalised to housekeeping genes) | miR-221 mimic | One-way analysis of variance (ANOVA) and Fisher’s *post-hoc* test |  | 3A |  |
|  |  |  | 1.541-fold | 0.008 |  | 0.003 |  | miR-221 inhibitor |  |  | 3B |  |
|  |  |  | 2.036-fold | 0.194 |  | 0.006 |  | miR-222 mimic |  |  | 3C |  |
|  |  |  | 1.300-fold | 0.142 |  | 0.025 |  | miR-222 inhibitor |  |  | 3D |  |
|  |  |  | 2.070-fold | 0.099 |  | 0.006 |  | miR-21 mimic |  |  | 3E |  |
|  |  |  | 1.513-fold | 0.139 |  | 0.010 |  | miR-21 inhibitor |  |  | 3F |  |
|  |  | CPEB3 mRNA | 1.520-fold | 0.059 |  | 0.015 |  | miR-21 mimic |  |  | 3G | |
| 4. Further verification of target genes | TIMP3 and CPEB3 were verified as targets of miR-21, and CPEB3 a target of miR-222 | TIMP3 | 1.28 | 0.347 | n = 3, N = 3 independent repeats | 0.030 | Luciferase activity | miR-222 mimic | One-way analysis of variance (ANOVA) and Fisher’s *post-hoc* test |  | 4A |  |
|  |  | CPEB3 | 0.728 | 0.263 |  | 0.010 |  | miR-222 mimic |  |  | 4B |  |
|  |  |  | 0.766 | 0.189 |  | 0.006 |  | miR-21 mimic |  |  |  |  |
|  |  | TIMP3 mRNA | 4.80-fold | 0.436 |  | <0.001 | arbitrary expression (normalised to housekeeping genes) | unloaded vs 7MPa |  |  | 4C |  |
